# Education and subjective well-being in Chinese rural population: A multi-group structural equation model

**DOI:** 10.1371/journal.pone.0264108

**Published:** 2022-03-10

**Authors:** Tianya Hou, Ruike Zhang, Yawei Xie, Qianlan Yin, Wenpeng Cai, Wei Dong, Guanghui Deng

**Affiliations:** Department of Psychology, Naval Medical University (Second Military Medical University), Shanghai, China; KDI School: KDI School of Public Policy and Management, UNITED STATES

## Abstract

**Purposes:**

This study aimed to explore the effect of education on subjective well-being (SWB) of Chinese rural dwellers who just shook off poverty in 2019 and to investigate the mediating role of social support and moderating role of age on the association.

**Methods:**

Social support rating scale (SSRS) and General Well-Being Schedule (GWBS) were administrated among 1094 Chinese rural dwellers from Anqing, Anhui Province, China. Structural equation modeling (SEM) and multi-group SEM were performed to examine the mediating role of social support and moderating role of age in the link between education and SWB, respectively.

**Results:**

The findings indicated that social support fully mediated the relationship between education and SWB in rural residents. Age moderated the indirect relationship between education and SWB (first stage moderation model) such that the effect of education on social support would be strengthened with aging process.

**Conclusions:**

The results add to our understanding of the protective role of education in SWB among Chinese rural dwellers, and shed new light on the potential mechanisms underlying the association between education and SWB with respect to the mediating role of social support and moderating role of age.

## Introduction

Research regarding subjective well-being (SWB) has flourished across the nation with the rapid development of economy and a substantial improvement of living standard in China [1]. Rural population takes an crucial part in the composition of Chinese population, accounting for nearly 46% of total Chinese in 2019 according to China’s system of household registration (known as hukou) [2]. More importantly, Chinese government plans to eradicate poverty in 2020 [3] and eliminating rural poverty is the key to achieve the goal. Therefore, more attention has been attached to rural population. Also, to further advance the industrialization and modernization inevitably requires enhancing SWB, especially for rural dwellers. A better understanding of the influencing factors and the underlying mechanisms is needed to enlighten the improvement of SWB. Although a handful of studies have investigated the determinants of SWB based on the rural population in China, most focused on the economic factors and no previous studies have studied the impact of educational attainment on the SWB in rural China [4, 5].

The globalization process has contributed to the radical changes of the critical role of higher education in social development [6]. Getting educated is usually perceived as the pursuit of better life [7] and attaining a higher level of education is expected to bring benefits to both individual and society [8–10], such as better health status, higher self-esteem, higher pay and social status and so on [9, 11]. Since these factors are verified to be correlated with SWB, a positive correlation is generally hypothesized between educational attainment and SWB. Several previous research has addressed the relationship, while the results are varied, inconsistent and contradicted between studies [9]. FitzRoy and Nolan have found a significant negative correlation between education and SWB based on the data from British Household Panel Survey (BHPS), while an equally significant positive correlation was reported with German Socio-Economic Panel (SOEP) data [12, 13]. Summarizing the previous research, the reported correlation between education and SWB are positive [14, 15], positive only at higher levels of education [16], positive only at moderate levels of education [17], insignificance [18] and negative [19]. However, education was often included as a control variable in the study of SWB, and the correlation between education and SWB is usually reported without detailed interpretation [9]. Many researchers call for more research on this issue [9, 20]. Thus, the association between education and SWB still needs more to verify.

The previous literature regarding this relationship is scarce and many researchers have appealed for further study in this area [9, 20, 21]. Powdthavee et al. has explored the role of education on SWB based on an equation model and presented that single-equation models might mislead the understanding with respect to the role of education in SWB. In addition, he stressed the influence of education on SWB is indirect through mediating variable such as health status, income level and so on [22]. Given that the majority of previous studies are confined to the urban population, the elder and university students, little is known concerning SWB of rural population even though they accounts for nearly half of the total population [23]. Thus, more attention should be paid to rural dwellers [24]. All these suggest the importance of exploring the intermediary variables underlying the association between education and SWB in Chinese rural residents. Although valuable contributions have been made by the previous research in the association between education and SWB, the potential underlying mechanisms concerning the mediator and moderator have largely remained unknown. To further understand how education associate with SWB, it is of great importance to explore the underpinning mediating and moderating mechanisms.

### Social support as a mediator

#### Education and social support

Social support as a highly modifiable variable refers to the emotional or material resources that a person perceives or receives to be available in one’s social network [25]. Higher levels of educational level have been demonstrated to be associated with high levels of social support. Existing literature provided robust evidence on the positive effects of graduating from a higher level of educational institutions on social support [26, 27]. Qi et al. reported a positive relationship between educational levels and social support among Chinese urban elderly people [28]. A recent study found Chinese family caregivers with higher educational levels reported higher levels of social support [27].

Çankaya and colleagues pointed out lower educational level might result in communication problems and failure to form social networks [26]. The relationship between education and social support could be also explained by the return to education. Education is viewed as one of the most important components of human capital [29]. The greatest contribution of Human Capital Management (HCM) theory is to reveal that the expenses of education are not only consumer spending but also productive investment [30]. Since education investment presents higher rate of return than the material capital investment [30], the rich bodies of domestic and overseas studies have focused on the return to education, especially on return to different levels of education. Gindling et al. reported that the return to higher education presented to be the highest based on the sample from Chinese Taiwan [31].

According to the concept of return to education, the type of return could be divided into two categories: tangible economic benefits and intangible spiritual benefits. The economic benefits entail the enhancement of financial gain and the improvement of living conditions [30], while the intangible benefits contain knowledge gain, promotion of individual quality and the upgrade of social status.

Individuals with higher levels of education attach great attention to mental health and have a strong desire to enhance psychological health. Thus, they are more likely to make better use of psychological service, which contributes to higher levels of emotional support [32]. In addition, Wang et al. has studied the relationship between social status and educational level in Xingjiang women and found that with one-year increase in the years of education, the average labour income increase 0.385%. Another article confirmed the positive correlation between educational level and social status [33]. The previous research presented robust evidence that the income and social status were positively correlated with perceived social support [34]. Higher educational level means better social-economic status, leading to more instrumental social support [35]. Thus, individuals with higher levels of education can be related to the higher levels of social support in rural population.

#### Social support and subjective wellbeing

Numerous previous studies have already confirmed the positive role of social support in SWB [36, 37]. A meta-analysis of 246 studies concerning the association between social support and SWB indicated a positive relationship [38]. Another meta-analysis conducted by Wang analyzed 21 studies and presented the significant correlation between social support and well-being indicators such as positive/negative mood state, depression and quality of life [39]. Apart from correlational studies, the experimental studies implying the causal relationship via animal research have also confirmed the positive impact of social support on SWB [38]. According to Cohen and Wills, social support exerted a positive influence on SWB since it could enhance the experience of positive mood, predictability in life and the sense of self-worth. Moreover, it could strengthen self-efficacy and self-esteem, which functions as a buffer when coping with stress [40].

In fact, a systematic review regarding the relationship between social support and subjective well-being has found the indirect association between educational level and subjective well-being via social support [36]. However, this finding was based on a sample of Americans and whether the result remains the same when it turns to Chinese rural dwellers is still unknown. In addition, despite the indirect association between education and SWB via social support has been studied, the research of such relationship is still rare and has not gained much attention, especially for rural population. Identifying the modifiable factor linking education and SWB could help inform public health policy and develop theoretically-grounded interventions to improve SWB among Chinese rural population.

### Age as a moderator

Although education may predict SWB through social support, the magnitude of the relationship among these three variables might vary across different conditions. Age, as a crucial demographic variable, may moderate the indirect pathway between education and SWB.

With aging process, people rely on different sources of social support to cope with life event stress. Levitt, Weber and Guacci found an age-related shift in social support network, with younger people relying more on friends and less on kinship in their support provision [41]. A study investigating age and social support perception also showed that levels of social support diminished with age, with friendship ties especially likely to decrease [42]. Consequently, the friendship ties become less important, while other social support resources such as instrumental support, emotional support from family ties and psychological service become increasingly important with getting older. As aforementioned, education could lead to higher social status and incomes, better use of social support, which in turns result in more instrumental and emotional social support. Thus, the association between education and social support become strengthened with aging process, which would further lead to the stronger association between education and SWB. Life-stage is a crucial variable affecting the indirect association between education and SWB through social support. Exploring the role of age in the association could contribute to a better understanding of the mechanisms and provide an insight for better applying interventions.

### The present study

**Fig 1:**
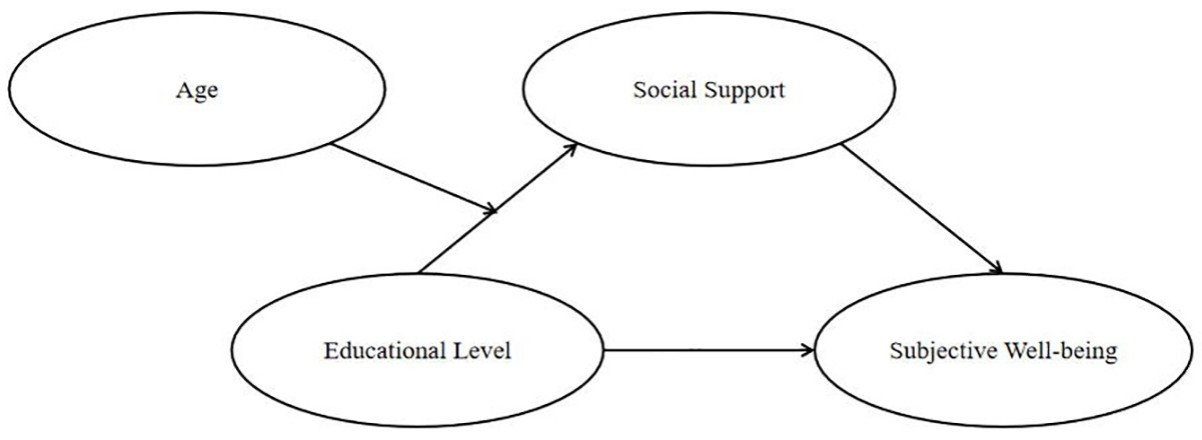
The proposed moderated mediation model.

In the current study, the aims were twofold: (a) to examine whether social support mediates the relationship between education and SWB in Chinese rural residents, and (b) to test whether the relationship between education and SWB via social support is moderated by age. A moderated mediation model is put forward to address the two research questions (see Fig 1). That is, how does education influence SWB and when is the association most potent? Based on the literature review, the present study had the following two hypotheses:

Hypothesis 1: Social support would have an indirect effect on the relationship between education and SWB in rural Chinese. To be specific, social support would be positively correlated with educational attainment, which in turn would be positively associated with SWB.
Hypothesis 2: Age would moderate the indirect path between education and SWB via social support such that the education-social support path would be stronger in higher age group compared with lower age rural residents. Given that we assume age would only moderate the first stage of the mediation process, the current study would call it “a first stage moderation model”.

## Method

### Participants, ethical approval and procedure

The research was approved by the ethics committee of Navy Medical University. The cross-sectional questionnaires were executed in the rural areas of Anqing city, Anhui Province, China. This area has just been lifted out of poverty in 2019 and the study was conducted in November, 2019. The online survey was distributed through local education department. Prior to the questionnaires, participants need to complete the informed consent, which claimed the participation was voluntary and they could withdraw at any time. A total of 1426 subjects filled in the questionnaires. After the exclusion of invalid data (i.e., the total seconds for filling out the questionnaire were less than 240s, missing values), 1094 participants were actually analyzed in the study, resulting in an effective response rate of 76.7%. Among the total participants, 45.3% are females and 88.2% participants were from complete original family. The participants reported a mean age of 39.77 (SD = 7.569). 23.7% of the respondents perceived their income to be above average.

### Measures

#### Subjective well-being

The General Well-Being Schedule (GWBS) was used to measure the subjective feeling of stress and well-being. It has been developed by Dupuy in the study conducted by the National Center for Health Statistics [43]. The multi-dimensional questionnaire consists of 18 items (e.g., “How happy, satisfied or pleased have you been with your personal life during the past month?”) [43]. It contains six dimensions: anxiety (items 2, 5, 8 and 16), depression (items 4, 12 and 18), positive well-being (items 6 and 11), self-control (items 3, 7 and 13), vitality (items 1, 9, 14 and 17) and general health (items 10 and 15). The scale has been translated into Chinese by Duan [44] with its validation. The GWBS has demonstrated enough internal consistency (the Cronbach’s alpha coefficients were 0.91 and 0.95 for male and female respectively) and adequate construct validity with the Pearson correlation coefficients ranging from 0.65 to 0.88. The first 14 items are scored from 1 to 6 or 1 to 5, while the remaining items use a 0–10 scales. Some items (items 1, 3, 6, 7, 9, 11, 15, and 16) need reversing scores. In the current study, the aspect of anxiety has a low correlation with other five aspects of SWB. When using latent variable in SEM, it requires at least medium correlation between the components of the latent variable. Thus, in this analysis, anxiety is not included.

#### Social support

Social support was measured by the Social Support Rating Scale (SSRS) developed by Xiao [45, 46]. The scale consists of 10 items, which contains three sub-scales measuring the subjective support (items 1, 3, 4 and 5), objective support (items 2, 6 and 7) and availability (items 8, 9 and 10) [46]. Liu et al. [47] has proved the scale has impressive validity and high reliability. The Pearson correlation coefficients between the total scores and the three sub-scale scores (range: 0.724~0.835) are higher than those between three sub-scales (range: 0.462~0.664) [47], which indicates both good content and construct validity. The Cronbach alpha coefficients were 0.896, 0.849, 0.825 and 0.833 for the total and sub-scales respectively [47].

#### Educational level

The participants were asked to self report the level of schooling (primary school, junior high school, senior high school, technical secondary school, junior college, university or upper and others (unschooled)). Then they were further categorized into lower educational level (primary school and others (unschooled)), medium educational level (junior high school) and high educational level (senior high school, technical secondary school, junior college and university or upper).

### Data analysis

Descriptive analysis was used to describe the variables of interest in the present study. The correlations between the study variables were performed by using the Statistical Package for Social Science (SPSS) 21.0 for windows. Statistical significance was established if a two-tailed p-value was smaller than 0.05.

Structural equation modeling (SEM) was performed by Amos 23.0 to test the mediation effect of social support on the relationship between educational level and subjective well-being in Chinese rural population. Several statistical indicators were used to determine the fitness of the model for SEM, including the goodness of fit index (GFI), Tucker-Lewis index (TLI), comparative fit index (CFI), incremental fit index (IFI), root mean square error of approximation (RMSEA), and the standardized root mean square residual (SRMR). According to the recommendation values from literature, the fitness is acceptable if the values of GFI, TLI, CFI and IFI are higher than 0.9 and the values of RMSEA and SRMR are lower than 0.08 [48, 49]. Two models were constructed to test the mediating effect. Model 1 examined the effect of education on SWB, while in model 2, social support was added on top of model 1 to test the mediating role of social support in the association between education and SWB. The significance of mediation effect was examined through bootstrap methods [50]. To be specific, the percentile bootstrap with 2000 bootstrap samples and the bias-corrected 95% confidence interval were performed in the present study. When the confidence interval does not include zero, it represents the presence of mediation effects [48, 51]. Multigroup SEM was used to examine the moderating effect of age. The analytical sample is split into two categories based on the moderator [52]. Kelley [53] has found that when using the values of 27% from the extreme scores as cut-off points, the division of upper and lower groups are optimal. We would compare the mediating effect in different groups. A two-tailed p-value for the comparison between groups smaller than 0.05 indicated the significant differences between groups.

## Results

The means, deviations, and the Pearson correlation coefficients were displayed in Table 1. The results presented that educational level was positively correlated with each dimension of social support, positive well-being, vitality and self-control, and negatively associated with age. Each dimension of social support was positively intercorrelated with each dimension of SWB, except the relation between objective support and anxiety. Age was positively related to self-control and negatively associated with availability.

**Table 1:** The mean, standard deviation and correlations between variables of interest.

	Mean	SD	1	2	3	4	5	6	7	8	9	10
1Positive well-being	6.761	2.000										
2General health	12.345	3.641	0.341**									
3Vitality	20.472	4.764	0.542**	0.420**								
4Depression	17.865	3.725	0.514**	0.410**	0.725**							
5Self-control	12.936	2.608	0.457**	0.297**	0.474**	0.467**						
6anxiety	16.464	3.296	0.109**	0.130**	0.025	0.060*	0.121**					
7Objective support	9.432	3.574	0.232**	0.121**	0.235**	0.170**	0.126**	0.058				
8Subjective support	26.316	4.976	0.327**	0.219**	0.318**	0.319**	0.218**	0.076*	0.352**			
9Availability	7.450	2.084	0.203**	0.162**	0.144**	0.129**	0.079**	0.072*	0.308**	0.279**		
10Educational level	1.906	0.661	0.089**	0.034	0.095**	0.017	0.119**	-0.048	0.112**	0.062*	0.122**	
11Age	39.620	8.026	0.004	0.009	0.021	0.042	0.097**	-0.007	0.044	0.049	-0.083**	-0.145**

Note: N = 1072,

*p<0.05,

**p<0.01

### Structural model

The first step for the structural model is to assess the path coefficients between the components, which is similar to the regression coefficients. Thus, theorized model with arrows suggesting exogenous and endogenous constructs or independent and dependent constructs was defined.

#### Testing mediation only

The first hypothesis assumed that the relationship between educational level and subjective well-being was mediated by the social support. The causal step procedures were used to test mediation [54]. From Table 2, it revealed that the mediation model met the criteria for acceptable fit, except for the significance of the χ^2^ test (*p*<0.05).

**Table 2:** Fit indices of proposed models.

Model	Model 1	Model 2
Chi-square (df)	151.134(20)	232.442(42)
Goodness-of-fit index (GFI)	0.965	0.962
Tucker-Lewis index (TLI)	0.909	0.902
Comparative fit index (CFI)	0.935	0.925
Incremental fit index (IFI)	0.935	0.926
Root mean square error of approximation (RMSEA)	0.077	0.064
Standardized root mean square residual (SRMR)	0.056	0.049

Model 1 assessed the impact of educational level on the subjective well-being

Model 2 included the mediation effect of social support on the relationship between education and SWB.

All models are adjusted for gender and perceived family economic status.

**Fig 2:**
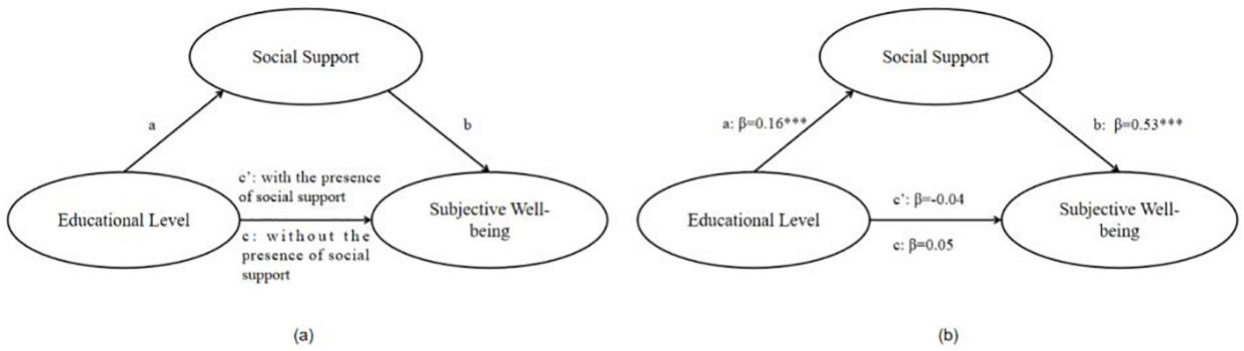
Path coefficients for mediation.

It is shown in Fig 2a and 2b that the path coefficients a and b in the current study were all significant while c´ was insignificant, which verified the mediation relationship.

The results from bootstrap methods for mediation effects presented that the bias-corrected 95% confidence interval for mediation relationship was from 0.042 to 0.133, which does not contain zero. This verified the mediation effects with at least 0.05 significance.

#### Moderated Mediation through multi-group SEM method

**Fig 3:**
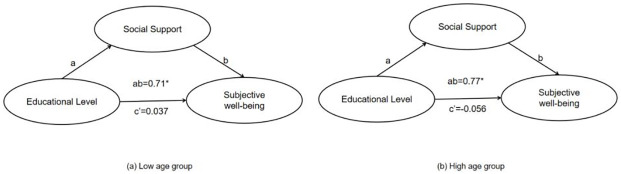
The relationship between educational level, social support and SWB in different age groups.

According to the multi-group SEM method, the sample should be divided into two groups based on age [52]. In the current study, 36 and 44 were used to divide the lower and upper age groups. The mediating effects were compared between the upper and lower age groups with the consideration of both indirect and direct paths. As shown in Fig 3a and 3b is used to represent the indirect path, while c’ indicates the direct effect.

**Fig 4:**
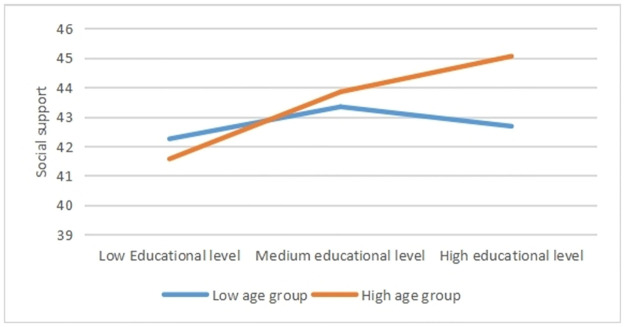
Moderating effect. Social support as a function of educational level and age. Functions are graphed for two levels of age. Note that this graph is only for descriptive purpose.

Bootstrapping of 2000 resampling with 95% biased-corrected confidence interval was performed to examine the significance of mediation in each group. From the Fig 3, although both low and high age groups present a full mediating effect, the indirect effect in high age group is stronger than that in low age group (p = 0.049). For descriptive purpose only, the current study plotted the relationship between educational level and social support, separately for low and high age groups (see Fig 4). Thus, the models in low and high age groups have exhibited significant different mediating effect, which verified the moderator role of age in the model.

## Discussion

Quantity of studies regarding the relationship between education and SWB has increased plentifully over the last two decades. However, the relationship stayed uncertain and the underlying mediating and moderating mechanisms remained largely unknown. The current study, used a sample of rural populations from the region where people have just shook off poverty in 2019 and proposed a moderated mediation model. The results verified the mediating role of social support and moderating role of age in the link between education and SWB.

### The mediating role of social support

The results presented a full mediation between education and SWB via social support. That is, educational level was positively related to social support, which in turn further positively predicts SWB. Thus, higher levels of social support can be one of the explanatory mechanisms for why rural dwellers with higher level of education are more likely to report better SWB. As far as I know, this is the first study to explore such relation in Chinese rural population.

Apart from the overall mediating mechanism, it is worth noting each of the separate stage in the mediation model. For the former part of the mediation process (education—social support), the current study confirms the notion that high educational attainment positively predicts more social support. This finding could be explained by the effect of return to education, which could be viewed as the capacity of a region to transform educational investment into human capital. Given that education is a progressive process, return to different levels of education would differ [55]. The study with an emphasis on the returns to education in China found returns to education are on the rise, while the disparity of return to different levels of education has gradually increased [55]. The gap between returns to high school/ technical secondary school and junior college or above has increased from 0.0076 in 1993 to 0.0291 in 2011 [55]. Li reported the overall tendency of returns to education continuously to ascend in rural China. The return to junior middle school or below education dropped, whereas the return to senior and above education was mounting up over years [56]. Thus, the disparity of return to different levels of education indicates that different educational level would result in different return to education. That is, the educational profit including more social support, higher social status, better living conditions and so on would differ according to different levels of education. Also the correlation results showed educational level was more correlated with objective social support and availability compared with subjective social support. This indicates that rural dwellers with higher educational level tend to get more actual social support and make better use of social support. These results are in line with the effect of return to education. Thus, people with higher educational attainment could get more social resources to get supported.

For the latter part of the mediation process (social support—SWB), this research revealed social support is positively associated with SWB. From the correlational result, each component of education is significantly correlated with each components of SWB (except the association between objective support and anxiety). This result is congruent with the direct and buffer effects of social support models [38, 40]. Social support exerts a direct impact on SWB since wide-range social network could provide positive experiences, a sense of self-worth, and a sense of stability and predictability. Also, it could help avoid negative events [40]. The stress-buffering model indicates that social support could buffer the influence of stressful events on the well-being. Also, the studies concerning coping with stress also regard social Support as one of the most useful approaches to deal with stress [40, 57]. Thus, social support would be a protective factor of SWB by buffering stress. The positive correlation is also in line with the previous studies including both correlational, experimental and prospective research, suggesting social support contributed to SWB [38, 40, 58–60].

### The moderating role of age

The second objective of this research was to explore whether age could moderate the indirect pathway between education and SWB (education-social support association). The results presented that age moderated the indirect relation between education and SWB (first stage moderation). The relationship between education and social support is strengthened with aging process. This could be explained by the role change with aging. Namely, there is an increasing trend in the possibility of reporting no close friendship with getting older and the changes in role account for a great proportion of the increase [61]. Thus, the proportion of different kinds of social support changes with aging process. Specifically, the effect of friendship, as a kind of social support source, decreased with aging due to retirement, death of friends, mobility difficulty and so on, while the influence of instrumental support and emotional support from family and psychological services became progressively more important as getting older. According to the return to education theory aforementioned, education could provide more social resources, broaden social network and make better use of psychological support resources [32, 40]. In sum, the social support that education provide is mainly economic benefits and spiritual benefits, whereas people rely more on the instrumental and emotional support from kin and mental service to get social support with ageing, which leads to the increase of the impact of education on social support as getting older. Therefore, consistent with our expectation, age moderated the indirect association between education and SWB (the first stage moderation).

### Strength and limitations

This research has both strengths and limitations. The strength contains the large sample size from rural China and robust statistical analysis (for instance: multigroup SEM analysis and bootstrapping). There are also some limitations that should be addressed. Firstly, this study is cross-sectional, which cannot infer causal relationship. Further scholars could adopt perspective or experimental study to explore. Secondly, the measure in the current study is questionnaire-based self-report which might threaten the internal validity. More objective indicators should be used in the further study, or integrating various kinds of methods for appraisal might decrease the influence of subjectivity.

### Implication

This study is, to the best of our knowledge, the first research to explore the mediating role of social support and the moderating role of age in the association between education and SWB, based on a sample of rural dwellers who has just been lifted out of poverty. This adds to our understanding of the impact of education on SWB in rural population and shed new light on the underlying mechanisms under the association. It has both vital theoretical and practical implications.

Firstly, these results highlight the role of education in SWB in rural China. Rural population accounts for nearly half of the total population. The study with emphasis on the SWB of rural people is of great importance in promoting the industrialization and urbanization in China. This study confirmed education is positively correlated with SWB in rural area, which indicated the popularization of education in rural region is useful to enhance SWB. The government should ensure more money and educational resources to be allocated to rural education. For example, some online classes could be freely accessed and shared.

Secondly, this study could help further understand the mediating role of social support in the association between education and SWB via establishing mediation model. It could provide insight in the intervention. Social support as a modifiable factor could be enhanced by public health initiatives aiming at providing social support resources including emotional support and instrumental support [62]. The local government in rural areas could establish psychological counseling centers to offer emotional support and improve their psychological resources. In addition, the government is anticipated to invest more money to support rural families in China.

Thirdly, this study suggests age as a moderator, which indicates that for older rural dwellers, to improve SWB via social support is more useful than younger people. Thus, older adults should be prioritized in terms of social support intervention.

## Conclusion

In summary, this study reveals education could be a protective factor in SWB based on Chinese rural dwellers who has just been lifted out of poverty. Moreover, the mediation analysis suggests social support can be a possible underpinning mechanisms. Furthermore, the moderated mediation model presents age moderated the association between education and social support, with the relation being stronger with aging. To be specific, improving the educational level of rural population could improve social support, especially for older rural dwellers.

## Supporting information

S1 Dataset The minimal anonymized data set necessary to replicate study findings. (SAV)

S1 Fig Statistical analysis of the mediation analysis. (DOCX)

S1 Appendix Social support rating scale. (DOCX)

S2 Appendix General well-being schedule. (DOCX)
