# Supplementary figures and images for: Education and subjective well-being in Chinese rural population: A multi-group structural equation model
Source: PLoS One. 2022 Mar 10;17(3):e0264108. doi: 10.1371/journal.pone.0264108 (PMC8912145; doi:10.1371/journal.pone.0264108)

**S1 Figure. Statistical analysis of the mediation analysis**
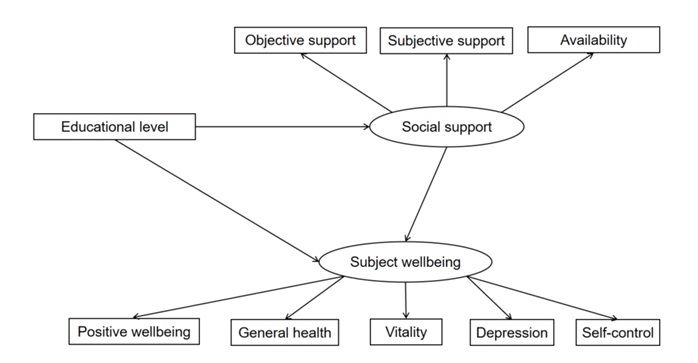

Supplement: S1 Fig — (DOCX) [file pone.0264108.s002.docx]
