# Supplementary material for: Education and subjective well-being in Chinese rural population: A multi-group structural equation model
Source: PLoS One. 2022 Mar 10;17(3):e0264108. doi: 10.1371/journal.pone.0264108 (PMC8912145; doi:10.1371/journal.pone.0264108)
Supplement: S1 Appendix — (DOCX) [file pone.0264108.s003.docx]

**S1 Appendix. Social support rating scale**

Please Choose the answer that best suits the situation[1].

**1. How many friends do you consider to be close enough to you that can count on them for help when you need it?**

1. None

2. 1 - 2

3. 3 - 5

4. 6 or more

**2. In the last year, you:**

1. Stayed away from others and lived alone

2. Moved a lot, and mostly lived with strangers

3. Lived with colleagues, friends, or classmates

4. Lived with your family

**3. You and your neighbors**

1. Never cared about each other

2. Showed some care when encountering difficulties

3. Some neighbors cared about you a lot

4. Most neighbors cared about you a lot

**4. You and your colleagues**

1. Never cared about each other

2. Showed some care when encountering difficulties

3. Some colleagues cared about you a lot.

4. Most colleagues cared about you a lot

**5. Support and care from family members (put a check mark where applicable)**

|  | None | Rare | Some support/care | Strong support/care |
| --- | --- | --- | --- | --- |
| Spouse | □ | □ | □ | □ |
| Parents | □ | □ | □ | □ |
| Children | □ | □ | □ | □ |
| Sisters or brothers | □ | □ | □ | □ |
| Other family members (e.g., sister-in-law, etc.) | □ | □ | □ | □ |

**6. In the past, when faced with an emergency, you have received financial or other material support from:**

1. None

2. The following (check all that apply):

a. Spouse

b. Other family members

c. Relatives

d. Friends

e. Colleagues

f. Employer

g. Union or government

h. Political or religious organizations, society, and nongovernmental organization

i. Other (please specify)

**7. In the past, when faced with an emergency, you have received console and other emotional support from:**

1. None

2. The following (check all that apply):

a. Husband or wife

b. Other family members

c. Relatives

d. Friends

e. Colleagues

f. Employer

g. Union or government

h. Political or religious organization, society, and nongovernmental organization

i. Other (please specify)

**8. When you feel sad or vexed, you.**

1. Never talk to anyone

2. Only talk to the closest one or two individuals

3. Will talk to friends if they ask

4. Will talk to friends even if they did not ask

**9. When you have difficulties/troubles, you.**

1. Count on yourself and do not accept help from others

2. Rarely ask for help

3. Sometime ask for help

4. Always look for help from family members, relatives, and organizations

**10. Your participation in activities organized by political or religious organizations, unions, and student associations, etc., can be described as follows:**

1. Never

2. Rarely

3. Frequently

4. Always and playing active roles in these activities

**References:**

1Xie RH, He G, Koszycki D, Walker M, Wen SW. Prenatal social support, postnatal social support, and postpartum depression. ANN EPIDEMIOL. 2009;19(9):637-43. 'doi:'10.1016/j.annepidem.2009.03.008.
