# Supplementary material for: Education and subjective well-being in Chinese rural population: A multi-group structural equation model
Source: PLoS One. 2022 Mar 10;17(3):e0264108. doi: 10.1371/journal.pone.0264108 (PMC8912145; doi:10.1371/journal.pone.0264108)
Supplement: S2 Appendix — (DOCX) [file pone.0264108.s004.docx]

**S2 Appendix. General well-being schedule**

1. How have you been feeling in general?

2. Have you been bothered by nervousness or your ‘nerves’?

3. Have you been in firm control of your behavior, thoughts, emotions, or feelings?

4. Have you felt so sad, discouraged, hopeless, or had so many problems that you wondered if anything is worthwhile?

5. Have you been under or felt you were under any strain, stress, or pressure?

6. How happy, satisfied, or pleased have you been with your personal life?

7. Have you had any reason to wonder if you were losing your mind, or losing control over the way you act, talk, think, feel, or of your memory?

8. Have you been anxious, worried, or upset?

9. Have you been waking up fresh and rested?

10. Have you been bothered by any illness, bodily disorder, pains, or fears about your health?

11. Has your daily life been full of things that were interesting to you?

12. Have you felt downhearted and blue?

13. Have you been feeling emotionally stable and sure of yourself?

14. Have you felt tired, worn out, used-up, or exhausted?

15. How concerned or worried about your health have you been?

16. How relaxed or tense have you been?

17. How much energy, pep, or vitality have you felt?

18. How depressed or cheerful have you been?

All items refer to a 1-month time frame. Items 1–14 are rated on a six-point Likert scale, while items 15–18 are rated from 0–10 [1].

**References:**

1Taylor JE, Poston II WSC, Haddock CK, Blackburn GL, Heber D, Heymsfield SB et al. Psychometric characteristics of the General Well-Being Schedule (GWB) with African–American women. QUAL LIFE RES. 2003;12(1):31-9. 'doi:'10.1023/A:1022052804109.
